# Relationship Between Medication Literacy and Frailty in Elderly Inpatients With Coronary Heart Disease: A Cross-Sectional Study in China

**DOI:** 10.3389/fphar.2021.691983

**Published:** 2021-07-08

**Authors:** Jiling Qu, Ting Zhou, Mengxin Xue, Huiping Sun, Yijing Shen, Yongbing Liu

**Affiliations:** School of Nursing, Yangzhou University, Yangzhou, China

**Keywords:** medication literacy, frailty, elderly, inpatients, coronary heart disease, relationship

## Abstract

**Background:** Mastering medication literacy may be related to medication safety, and the identification of frailty is very important for the prognosis of coronary heart disease (CHD). Few studies have examined the relationship between medication literacy and frailty in patients with CHD. The aim of this study was to investigate the state of medication literacy and frailty in patients with CHD and to explore the relationship between medication literacy and frailty.

**Methods:** A cross-sectional investigation evaluated 295 inpatients with CHD recruited from hospitals in Yangzhou, China. Demographic and clinical data on participants were collected using a general information questionnaire. The Chinese medication literacy scale was used to evaluate medication literacy. The Fried Frailty Phenotype scale was used to evaluate frailty. Univariate analysis employed chi-square test and Kruskal-Wallis H test to examine the potential factors affecting frailty. Taking frailty status as the outcome variable, the ordered logistic regression model was used to analyze the relationship between the degree of medication literacy and frailty. Spearman’s correlation analysis was used to analyze the correlation between medication literacy and frailty.

**Results:** A total of 280 elderly CHD inpatients were included in the analysis. There were 116 (41.4%) individuals with inadequate medication literacy and 89 (31.8%) frail individuals. Ordered logistic regression analysis showed that the age (*p* < 0.001, *OR* = 1.089), Charson Comorbidity Index (*p* = 0.029, *OR* = 1.300), number of medications taken (*p* = 0.012, *OR* = 1.137), and medication literacy (*p* < 0.05, *OR >* 1) were independent predictors of debilitating risk factors. The population with inadequate medication literacy had a 2.759 times greater risk of frailty than adequate medication literacy (*p* < 0.001, *OR* = 2.759); The population with marginal medication literacy had a 2.239 times greater risk of frailty than adequate medication literacy (*p* = 0.010, *OR* = 2.239). Spearman’s correlation analysis showed that the medication literacy grade was associated with the frailty grade in elderly CHD patients (*R* = -0.260, *p* < 0.001).

**Conclusion:** The study showed a significant correlation between medical literacy and frailty in patients with CHD. The results suggested that medication literacy was an important consideration in the development, implementation, and evaluation of frailty.

## Introduction

Frailty is defined as a series of syndromes caused by a decreased physiological reserve, such as decreased body function and chronic diseases (Schoufour et al., 2017). It seriously affects the health status and increases the risk of falls, fractures, infections, suicide, disability, and death among older people (Cunha et al., 2019; Houghton et al., 2020; Kurobe et al., 2021). The prevalence of frailty ranges from 10 to 60% in older adults in cardiovascular care (Afilalo et al., 2014). Frailty is an independent prognostic marker of the composite of mortality, reinfarction, and mortality in patients aged ≥75 years admitted due to myocardial infarction (Alonso Salinas et al., 2018). Studies have shown that higher aging trajectories in frailty scores were associated with elevated risks for cardiovascular, other-cause, and all-cause death among older Japanese individuals receiving health checkups (Taniguchi et al., 2020). Frailty is reversible, but requires intervention. A recent review and meta-analysis have shown that only 3% of frail older people spontaneously reverted to a robust state at a later date (Rodriguez-Mañas and Fried, 2015; Kojima et al., 2019). In order to reduce the incidence of death and complications in patients with coronary heart disease (CHD), it is essential to screen for frailty in a timely fashion, find the influencing factors of frailty in patients with CHD, and carry out an effective intervention according to these factors (Kang et al., 2015).

Patients with CHD usually require oral medications to achieve and maintain effective symptom control and prevent disease progression (Zhong et al., 2016). Good medication literacy is the premise of ensuring drug use safety (Li et al., 2020). The term “medication literacy” first appeared in a government document of the Committee of the Regulatory Agency for Medicines Safety and Healthcare Products in the United Kingdom in 2005. It referred to health literacy as “a series of skills required to obtain, understand and use drug information” (Shen et al., 2018). Pharmacy practices and laws vary widely around the world. In order to help healthcare workers around the world realize the importance of medical literacy in drug use, Pouliot et al. (Pouliot et al., 2018) consulted international experts using the Delphi method and proposed an expert consensus on the concept of medication literacy, which refers to the ability of individuals to obtain, understand, communicate, calculate, and process specific drug information and make informed drug treatment and health decisions in order to achieve safe and effective drug use. Research has shown that low health literacy is associated with frailty (Hou, 2019), and medication literacy is the embodiment of health literacy in the field of medicine (Raynor, 2009). Frailty is associated with an increased susceptibility to adverse drug events and drug-related injuries (Liau et al., 2021). However, there is no direct evidence of a link between medication literacy and frailty.

Therefore, the purpose of our study was to investigate the relationship between medication literacy and frailty in elderly patients with CHD in order to describe a new and targeted intervention problem for healthcare personnel, improve the quality of patient life, and reduce the risk of complications.

## Materials and Methods

### Design, Setting, and Participants

The study was approved by the Ethics Review Committee of the School of Nursing, Yangzhou University (Ethical Batch Number: YZUHL20200012). A cross-sectional survey was conducted in a cardiology ward of a tertiary hospital in Yangzhou, Jiangsu Province, China between August 2020 and January 2021. The convenient sampling method was used to extract the research subject data.

Subjects were eligible if they met the following inclusion criteria: 1) age ≥60 years and good communication skills; 2) met the diagnostic criteria of coronary atherosclerotic heart disease of the American Heart Association; and 3) provided informed consent and voluntarily participated in the study. Patients were excluded if they had any of the following conditions: 1) acute or terminal stage of a disease, severe cardiopulmonary and renal insufficiency; 2) patients with grade IV cardiac function; and 3) engaged in healthcare-related work currently or before retirement.

Our study is a cross-sectional study, according to the cross-sectional sample size calculation formula, which is: *N* = Z^2^_*α*/2_P (1-P)/d^2^. According to existing studies (Hou et al., 2019), the frailty incidence (*P*) of elderly hospitalized patients with CHD evaluated by the fried frailty phenotype scale was 20.8%. In our study, we set *α* = 0.05, *Z*_*α*/2_ = 1.96, allowable error (d) = 5%; After calculation, the required sample size was 253 cases. Considering that invalid questionnaires constitute 10% of the total cases, the required sample size was 278 cases. A total of 295 questionnaires were sent out in the present study, and 15 invalid questionnaires were eliminated. Thus, a total of 280 valid questionnaires were finally recovered, with an effective recovery rate of 94.9%.

### Survey Procedures

Inpatients anonymously filled out questionnaires after signing the informed consent form. The survey was conducted *via* one-to-one and face-to-face interviews. The interviewees were all postgraduate students from School of Nursing, Yangzhou University who had received similar training. The same assistive tools were used to measure frailty and medication literacy. If the respondents were illiterate or unable to fill in the forms by themselves, the investigators read the questionnaire to them and assisted them in completing it. After the questionnaire was completed, the researcher checked and retrieved it immediately. If there were omissions or obvious mistakes, the researcher assisted the patient in correcting them.

### Data Collection

#### General Condition Questionnaire

The investigators designed a self-administered general condition questionnaire, which included questions about age, sex, body mass index (BMI), education level, marital status, economic status, smoking status, drinking status, Charson Comorbidity Index (CCI), and number of medications taken. The patients who were unsure of their height and weight were measured on site. The medication number inquiry was as follows: How many drugs did you take for more than 3 months before hospitalization?

#### Chinese Version of the Medication Literacy Scale

The Chinese Version of the Medication Literacy Scale was used to evaluate medication literacy. This scale was originally developed by Sauceda et al. (2012) from the University of Texas at El Paso in the United States. Zheng et al. (2016) sinicized the English version of the scale. The retest reliability of the Chinese scale was 0.885 and the sub-half reliability was 0.840. The correlation coefficient between each item and the total score of the scale was 0.427–0.587. The scale was composed of four simulated drug use scenarios containing 14 items and was scored on a two-point scale (1 point for correct answers and 0 points for wrong answers). The score for each item was added to the total score of the questionnaire. The higher the score, the higher the patient's level of medication literacy. Patients with scores >10 were considered to have “adequate medication literacy”. Those with scores of 4–10 were considered to have “marginal medication literacy”, whereas patients with scores <4 were considered to have “inadequate medication literacy”. This scale is mainly used to measure the ability of patients to read, understand, calculate, and deal with drug-related problems in the medical information environment in order to evaluate the level of their medication literacy.

#### Fried Frailty Phenotype Scale

The frailty assessment was based on the Fried Frailty Phenotype Scale proposed by Fried et al. (Fried et al., 2001) at the School of Medicine at Johns Hopkins University in 2001. There are five items on the scale, including weight loss, slowness, weakness, low physical activity, and exhaustion.

1) Weight loss: It is an unintentional loss of ≥4.5 kg or a loss of ≥5% body weight in the past 1 year.
2) Slowness: The time required to walk 4.6 m at a normal speed was used as an indicator of slowness. Slow walking speed was defined as ≥6 s when a male is >173 cm in height and a female is >159 cm in height or 7 s when a male is ≤173 cm in height and a female is ≤159 cm in height.
3) Weakness: Hydraulic dynamometer was used to measure grip strength as an indicator of weakness. Older adults in a sitting position used the dominant hand to grip an object three times and the researcher recorded the maximum value. Criteria proposed by Fried et al. (2001) was used to define the weakness.
4) Low physical activity: The International Physical Activity Questionnaire was used to assess physical activity (Liou et al., 2008); Males who expended <383 kcal/w and females who expended <270 kcal/w were considered to have low physical activity.
5) Exhaustion: Poor endurance and energy were assessed using the depression scale, specifically, to check whether the answer to either of these questions is yes: “Last week, I felt like everything I did needed an effort”; “I can’t walk forward”. If positive response was given to either of these questions, the participant was thought to be exhausted.

Each item scored one point if it was present. Otherwise, no points were scored. Patients with scores ≥3 were considered as “frail”, those with scores of one to two were considered as “pre-frail”, and patients with a score of 0 were considered as “not frail”. This scale is easy to evaluate objectively and it is widely used. In the present study, Cronbach’s *α* coefficient of the questionnaire was 0.671.

### Statistical Analysis

Data were analyzed using SPSS (version 26.0, Chicago, IL, United States) software. A *p*-value of < 0.05 was considered statistically significant.

Descriptive statistical methods were used to describe the inpatient’s baseline characteristics, level of medication literacy, and frailty. Univariate analysis used the chi-square test and Kruskal-Wallis H test to examine the influencing factors of frailty. The frailty status was used as the outcome variable to conduct the ordered multi-classification logistic regression analysis for multivariate analysis. Spearman’s correlation analysis was used to analyze the correlation between medication literacy and frailty.

## Results

### General Characteristics of Participants

A total of 280 elderly CHD inpatients were included in the analysis. The characteristics of the inpatients with CHD are shown in Table 1. The study included 137 (48.9%) males and 143 (51.1%) females. The median patient age was 73.0 (68.0–79.0) years. The median number of drugs used in the patients was 3.0 (1.0–5.0).

**TABLE 1:** | Characteristics of inpatients with CHD (*N* = 280).

Variable name	—	No. of participants (*N* = 280)	Percentage (%)
Sex	Male	137	48.9
—	Female	143	51.1
Education (year)	≤9	207	73.9
—	≥10	73	26.1
Marital status	Free	201	71.8
—	Unaccompanied	79	28.2
Monthly income	<2000	113	40.4
—	2000–5,000	112	40.0
—	>5,000	55	19.6
Smoke	No	170	60.7
—	Yes	110	39.3
Drink	No	174	62.1
—	Yes	106	37.9

### Chinese Version of the Medication Literacy Scale

The medication literacy of elderly patients with CHD is shown in Table 2. The median medication literacy score was 2.0 (0.0–3.0). There were 116 (41.4%) people with inadequate medication literacy, 70 (25.0%) with marginal medication literacy, and 94 (33.6%) with adequate medication literacy. The highest accuracy of item 9 was 165 (58.9%), and the lowest accuracy of item 11 was 76 (27.1%).

**TABLE 2:** | Medication literacy for inpatients with CHD (*N* = 280).

Items	No. of participants who answered correctly (*n* = 280)	Percentage (%)
Case scenario 1	—	—
1 According to the label, how many times a day should your mother inject the medicine?	157	56.1
2 Please show me how much medicine you should put into the syringe in the morning and mark the amount on the syringe	124	44.3
3 According to the instructions, please tell us or point out where the three parts of the body where your mother can inject the medicine are?	106	37.9
4 According to the instructions, please tell me what is the right angle at which you should inject the medicine?	95	33.9
5 Looking at the prescription, if your mother’s medicine runs out, where should you get a new prescription?	130	46.4
Case scenario 2	—	—
6 Looking at the instructions on this box, what is the dose of the medicine you should give to your niece?	137	48.9
7 If you know the medicine dosage that your niece needs to take, please mark on the cup up to what line you should pour the medicine	118	42.1
8 According to the directions, what is the maximum dosage your niece should take?	110	39.3
Case scenario 3	—	—
9 Looking at this prescription, what is the name of the medicine that you need to buy at the pharmacy?	165	58.9
10 According to the prescription, how many pills should you take?	112	40
11 Looking at this bottle, the medicine in the bottle has a similar purpose compared to the medicine on the prescription. If you need to take 30 pills to treat the infection, how many boxes should you buy to have the correct amount of antibiotic required by the original prescription?	76	27.1
Case scenario 4	—	—
12 Looking at the box, when does the medicine go out of date?	143	51.1
13 According to the directions, what is or what are the active ingredients in each pill?	145	51.8
14 Please look carefully at the box. For what reason should you stop taking the medicine?	133	47.5

### Fried Frailty Phenotype Scale

The frailty of elderly patients with CHD is shown in Table 3. The median frailty score was 6.0 (0.0–12.7). There were 80 (28.6%) patients who were not frail, 111 (39.6%) who were considered pre-frail, and 89 (31.8%) frail individuals. The highest satisfaction for item 3 was 167 (59.6%), and the lowest satisfaction for item 1 was 43 (15.4%).

**TABLE 3:** | Fried for inpatients with CHD (*N* = 280).

Items	No. of participants (*n* = 280)	Percentage (%)
1 Weight loss	43	15.4
2 Slowness	96	34.3
3 Weakness	167	49.6
4 Low physical activity	79	28.2
5 Exhaustion	99	35.4

### Associated Factors of Frailty in Elderly Patients With CHD

Results for univariate analysis of frailty determinants for inpatients with CHD are shown in Table 4. A total of four factors were significantly associated with frailty. Compared to the population with adequate medication literacy, those with marginal medication literacy and inadequate medication literacy were more likely to be in a frail state (*p* < 0.001). Older patients (*p* < 0.001), those with a higher CCI (*p* < 0.001), and individuals who used more drugs (*p* < 0.001) were more likely to be in a frail state.

**TABLE 4:** | Results of univariate analysis of frailty determinants for inpatients with CHD (*N* = 280).

Variable name	—	Number of cases (Percentage %)			*χ*2*/H*	*P*
	—	Not frail (*n* = 80)	Prefrail (*n* = 111)	Frail (*n* = 89)		
Age	—	68.5 (64.0–72.0)^a^	73.0 (69.0–79.3)^a^	78.0 (73.0–84.0)^a^	68.021^b^	<0.001
Sex	Male	44 (32.1)	57 (41.6)	36 (26.3)	4.002^c^	0.135
	Female	36 (25.2)	54 (37.8)	53 (37.1)	—	—
BMI	—	24.1 (22.2–30.0)^a^	24.2 (22.2–25.7)^a^	23.8 (21.1–27.2)^a^	0.393^b^	0.822
Education (year)	≤9	52 (25.1)	87 (42.0)	68 (32.9)	4.732^c^	0.094
	≥10	28 (38.4)	24 (32.9)	21 (28.8)	—	—
Marital status	Yes	64 (31.8)	76 (37.8)	61 (30.3)	3.731^c^	0.158
	No	16 (20.3)	35 (44.3)	28 (35.4)	—	—
Monthly income	<2000	29 (25.7)	47 (41.6)	37 (32.7)	1.236^c^	0.874
	2000–5,000	35 (31.3)	44 (39.3)	33 (29.5)	—	—
	>5,000	16 (29.1)	20 (36.4)	19 (34.5)	—	—
Smoke	No	45 (26.5)	67 (39.4)	58 (34.1)	1.415^c^	0.493
	Yes	35 (31.8)	44 (40)	31 (28.2)	—	—
Drink	No	42 (24.1)	70 (40.2)	62 (35.6)	5.341^c^	0.069
	Yes	38 (35.8)	41 (38.7)	27 (25.5)	—	—
CCI	—	1.5 (0–3)^a^	3 (2–5)^a^	4 (2–6)^a^	32.336^b^	<0.001
Number of medications	—	3 (2–3)^a^	4 (3–5)^a^	4 (4–5)^a^	53.562^b^	<0.001
Medication literacy	Inadequate	21 (18.1)	49 (42.2)	46 (39.7)	22.289^c^	<0.001
	Marginal	18 (25.7)	25 (35.7)	27 (38.6)	—	—
	Adequate	41 (43.6)	37 (39.4)	16 (17)	—	—

a Notes: median (IQR)

b Kruskal-Wallis H test

c chi-square test.

The frailty grade (frailty, pre-frailty, and non-frailty) was taken as the dependent variable. The age, CCI, number of medications taken, and medication literacy were used as the independent variables. Ordered logistic regression analysis was then conducted. Table 5 represents the results of logistic regression analysis for frailty determinants for inpatients with CHD. The results showed that the age (*p* < 0.001, *OR* = 1.089), CCI (*p* = 0.029, *OR* = 1.300), number of medications taken (*p* = 0.012, *OR* = 1.137), and medication literacy (*p* < 0.05, *OR* > 1) were independent predictors of debilitating risk factors. The population with inadequate medication literacy had a 2.759 times greater risk of frailty than adequate medication literacy (*p* < 0.001, *OR* = 2.759); The population with marginal medication literacy had a 2.239 times greater risk of frailty than adequate medication literacy (*p* = 0.010, *OR* = 2.239).

**TABLE 5:** | Results of logistic regression analysis of frailty determinants for inpatients with CHD (*N* = 280).

Effect		*β*	SE	Wald	*P*	*OR*	95%CI
Age	—	0.085	0.021	16.170	<0.001	1.089	0.044 ∼ 0.126
CCI	—	0.262	0.120	4.777	0.029	1.300	0.027 ∼ 0.498
Number of medications	—	0.128	0.051	6.366	0.012	1.137	0.028 ∼ 0.227
Medication literacy	Inadequate	1.015	0.283	12.886	<0.001	2.759	0.461 ∼ 1.569
	Marginal	0.806	0.316	6.495	0.011	2.239	0.186 ∼ 1.425
	Adequate	—	—	—	—	—	—

### Correlation Between Frailty and Medication Literacy in Elderly Patients With CHD

Spearman’s correlation analysis of the medication literacy grade and frailty grade in elderly CHD patients showed that the medication literacy grade was associated with frailty grade in elderly CHD patients (*R* = −0.260, *p* < 0.001), which was statistically significant.

## Discussion

This population-based cross-sectional study described the medication literacy and frailty in a group of Chinese inpatients with CHD and explored the correlation between medication literacy and frailty.

Based on the analysis results, 107 people (44.03%) had inadequate medication literacy, 59 people (24.28%) had marginal medication literacy, and 77 people (31.69%) had adequate medication literacy. The incidence of inadequate medication literacy in this study was higher than the 20.0% in the Zheng et al. study (Zheng et al., 2019). The reasons for this may be related to age. The subjects of the present study were the elderly (≥60 years old), while those in the Zheng et al. study were adults ≥18 years old. This suggested that the levels of medication literacy among the elderly was more worrying. Memory and comprehension gradually decrease with age due to deterioration of the physical functions. In addition, the elderly hold relatively traditional views and their ability to accept new things is weak. Therefore, the knowledge and skills related to drug use are insufficient, and the level of medication literacy is low. It has been suggested that medical staff should pay attention to drug education of the elderly in clinical practice (Hao, 2018). The use of more intuitive charts or concise wording can also encourage family members to participate in medication management in order to improve the elderly patients’ medication literacy.

Using correlation analysis, the present study found that the level of medication literacy was negatively correlated with the state of frailty (*R* = −0.260, *p* = 0.001). It is consistent with the research results by Liu et al. (2020). That is, the higher the level of medication literacy, the lower the degree of frailty. Ordered logistic regression analysis found that medication literacy was an independent predictor of frailty. This is consistent with Uemura et al. research results (Uemura et al., 2021). This may be because it is difficult for patients with inadequate medication literacy to understand information related to medication, and they cannot effectively cooperate with treatment directions, which is more likely to lead to health status decline and then the occurrence of frailty. Therefore, attention should be paid to medication literacy to reduce the risk of debilitating status deterioration in patients with CHD with limited drug knowledge. Secondary CHD prophylaxis usually includes antiplatelet agents (aspirin adenosine diphosphate receptor antagonists clopidogrel or ticagrelor), angiotensin-converting enzyme inhibitors or angiotensin receptor antagonists, statins, beta-blockers, and nitrates (Szummer et al., 2017). If patients suffer from other diseases, the number of treatment drugs increases. Thus, CHD patients often face the problem of multiple drug use (Duan and Jing, 2019). Adequate medication literacy makes it easier for patients to obtain correct drug information, maintain good medication habits, and effectively cooperate with treatment, which is more conducive to maintaining good health status, thus reducing the probability of frailty. Medication literacy programs can be used to achieve this goal (Shen et al., 2019). Health lectures, group discussions, personalized consultation, demonstration of teaching skills, practical exercises, automatic reminders, medication boxes, and medication cards can be utilized to comprehensively improve patients’ literacy in all aspects of medication knowledge, attitude, skills, and behaviors.

Besides medication literacy, age, CCI, and number of medicines have been reported to be significant determinants using logistic regression. These results are consistent with the findings from previous studies (Vetrano et al., 2019; Xu et al., 2021; Palmer et al., 2019). Therefore, an integrated multifaceted approach is needed to improve frailty in patients with chronic disease.

The present study had the following advantages: The findings showed that medication literacy is related to frailty, whereas most of the previous studies have concentrated on the relationship between health literacy and frailty. The results of the present study will improve the understanding of the impact of medication literacy on health status. There were some limitations in this report. First, this study had a cross-sectional design, which could only explain the correlation between medication literacy and frailty in patients with CHD, but could not prove a causal relationship. In subsequent studies, follow-up will be added to dynamically observe the effect of medication literacy on frailty. Second, this study was conducted in a tertiary hospital in China, and the results may not be representative. More multicenter cohort studies with a larger sample size should be conducted.

## Conclusion

The study showed that there was an association between medication literacy and frailty in patients with CHD. Medication literacy was an important consideration in the development, implementation, and evaluation of frailty. The study also provided preliminary information for the development of effective healthcare interventions.

## Data Availability

The original contributions presented in the study are included in the article/Supplementary Material, further inquiries can be directed to the corresponding author.
